# The Collection of Garbage Again

**Published:** 1900-09

**Authors:** 


### THE COLLECTION OF GARBAGE AGAIN.

THE article on the collection of garbage, published in these columns last month, aroused a great deal of public interest which took the form of a widespread action looking to the abatement of the nuisance. The daily newspapers took the matter up and the demand for a change in the collection system was general. A petition was begun and within a very short time something like 13,000 names were presented to the Board of Public Works, which with the interests of the city in view, presented the whole matter to the Board of Aldermen, with a recommendation that a change be made at once. The members of the Board of Aldermen, with that utter disregard for public affairs in which they have no personal or pecuniary interest, took the matter up and after looking it over, calmly sent it to a committee where it will remain until the Board goes into session again after the summer vacation. This is the usual method when the board desires time to either put on the screws or to look into a public matter with a view to promoting their own interests.

Whatever may have been the object of the board in taking the action it did in this matter, the fact remains that the question is one not of personal profit, but one of public health, and the sooner the eminent financiers on the board of aldermen learn to separate financial and sanitary matters the better it will be for the city's interests. A patent fact in connection with the juggling of the proposed change is that the men who were most anxious and fought the hardest for reference to committee, were the men who were members of that committee to which the whole matter was referred. To those versed in municipal matters there is but one construction to be placed on this action. Before the matter can be acted upon the committee must report back to the board. If the report is unfavorable that ends it for the time being, so far as that committee is concerned. And it is notorious that aldermanic committees do not make favorable reports on matters in which contractors are interested until they have had "hearings" which may be interpreted any way one wishes, and according to one's familiarity with the methods of the Board of Aldermen.

*En passant*, it might be remarked that just previous to the days of the Broadway Railroad's connection with the New York Board of Aldermen there were several of these "hearings" and shortly afterward there was a wholesale exposure and those of the members of that Board of Aldermen who did not suddenly move to Canada transferred their spheres of usefulness from the City Hall to the State Prison at Sing Sing under sentence for "boodling."

The daily newspapers deserve the thanks of the people of Buffalo for the unanimity of action in the garbage matter and there is every indication that when the Board of Aldermen gets together again they will have had time to read and digest the newspaper criticisms of their action and be prepared to declare themselves. They should consider carefully and deliberately the statement made in more than one of the Buffalo newspapers and on more than one occasion that the mode of procedure looked, to say the least, "suspicious;" yet this board has been under suspicion so often of late that it is apparently becoming used to it and will probably pay little attention to existing public feeling until the weight of suspicion crushes them. There is such a thing.

---

